# Neonatal asphyxia as an inflammatory disease: Reactive oxygen species and cytokines

**DOI:** 10.3389/fped.2023.1070743

**Published:** 2023-01-27

**Authors:** Kaoru Okazaki, Shinji Nakamura, Kosuke Koyano, Yukihiko Konishi, Masatoshi Kondo, Takashi Kusaka

**Affiliations:** ^1^ Department of Neonatology, Tokyo Metropolitan Children's Medical Center, Tokyo, Japan; ^2^ Department of Pediatrics, Faculty of Medicine, Kagawa University, Kagawa, Japan; ^3^ Maternal Perinatal Center, Faculty of Medicine, Kagawa University, Kagawa, Japan

**Keywords:** asphyxia, ROS, HMGB1, NF-κB, cytokines, neutrophils, TLRs, neonate

## Abstract

Neonatologists resuscitate asphyxiated neonates by every available means, including positive ventilation, oxygen therapy, and drugs. Asphyxiated neonates sometimes present symptoms that mimic those of inflammation, such as fever and edema. The main pathophysiology of the asphyxia is inflammation caused by hypoxic-ischemic reperfusion. At birth or in the perinatal period, neonates may suffer several, hypoxic insults, which can activate inflammatory cells and inflammatory mediator production leading to the release of larger quantities of reactive oxygen species (ROS). This in turn triggers the production of oxygen stress-induced high mobility group box-1 (HMGB-1), an endogenous damage-associated molecular patterns (DAMPs) protein bound to toll-like receptor (TLR) -4, which activates nuclear factor-kappa B (NF-κB), resulting in the production of excess inflammatory mediators. ROS and inflammatory mediators are produced not only in activated inflammatory cells but also in non-immune cells, such as endothelial cells. Hypothermia inhibits pro-inflammatory mediators. A combination therapy of hypothermia and medications, such as erythropoietin and melatonin, is attracting attention now. These medications have both anti-oxidant and anti-inflammatory effects. As the inflammatory response and oxidative stress play a critical role in the pathophysiology of neonatal asphyxia, these drugs may contribute to improving patient outcomes.

## 1. Introduction

Neonatal asphyxia (NA) is caused by respiratory and/or circulatory failure *in utero* or at birth, as may be seen in uterine rupture, placental abruptio, and umbilical cord prolapse (1). Thus, the pathophysiology of NA is associated with ischemic reperfusion (IR) injuries, which cause oxidative stress and excessive systemic inflammatory response syndrome (SIRS). First, hypoxia-ischemia decreases the oxygen supply and depletes adenosine triphosphate to cause primary injuries, such as cell swelling or death, in addition to oxidative stress. Second, reperfusion causes latent injuries, such as pro-apoptotic signaling, N-methyl-D-aspartate receptor hyperexcitability, inflammation, and the production of oxygen-related species.

Neonatal asphyxia is caused by respiratory and/or circulatory failure *in utero* or at birth, as may be seen in uterine rupture, placental abruptio, and umbilical cord prolapse (1). Thus, the pathophysiology of neonatal asphyxia is associated with ischemic reperfusion injuries, which cause oxidative stress. First, hypoxia-ischemia decreases the oxygen supply and depletes adenosine triphosphate to cause primary injuries, such as cell swelling or death, in addition to oxidative stress. Second, reperfusion causes latent injuries, such as pro-apoptotic signaling, N-methyl-D-aspartate receptor hyperexcitability, inflammation, and the production of oxygen-related species.

Hypoxia-ischemia in neonatal asphyxia is resolved using oxygen therapy, positive ventilation, chest compression or drugs. However, a sudden increase in the supply of oxygen and blood can exacerbate oxidative stress. The secondary energy failure then causes more severe neuronal dysfunction *via* mitochondrial collapse, cell death, seizure, inflammation, and oxidative stress. The latter two are closely interlinked during neonatal asphyxia. The main role of inflammation is to protect tissues and organs from injuries and to prevent unfavorable factors from flowing into tissue locally or systemically *via* thromboembolisms caused by hypercoagulation. Inflammatory immune cells, such as leukocytes, immediately infiltrate the damaged region, strengthen inflammation, remove the stimulus, and repair the tissue (2). Inflammation is characterized by five signs, including pain, heat, redness, swelling, and loss of function. Asphyxiated neonates sometimes present symptoms that mimic those of inflammation, such as fever and edema. Hypoxia is the main cause of inflammation. A general, inflammatory, defensive reaction to hypoxia or ischemia-reperfusion damage incurred during delivery is associated with neonatal asphyxia. In the present review, we describe the relationship between inflammatory mediators and oxidative stress in neonatal asphyxia.

## 2. Oxidative stress due to reactive oxygen species (ROS) in neonatal asphyxia

The environment of the neonatal brain conduces to Reactive oxygen species (ROS) production. The neonatal brain includes plenty of lipids and iron (Fe^2+^) (3). These lipids, which are necessary for myelination, may generate free radicals *via* peroxidation. Iron is associated not only with growth and development, but also with the generation of ROS, such as hydroxyl radicals, through the Fenton reaction. Moreover, both lipids and iron are related to ferroptosis, an iron-dependent, non-apoptotic form of cell death (4, 5). The neonatal brain is more sensitive to ROS than the brain of older individuals.

ROS, including the superoxide anion (•O_2_^−^), hydrogen peroxide (H_2_O_2_), and hydroxyl radical (•OH), play a central role in oxidative stress. In particular, mitochondrial ROS (mit-ROS) is associated with neonatal brain injury caused by hypoxia-ischemia and hypoglycemia (6–8). The mitochondria are a major source of ischemic reperfusion-induced ROS but stores a large amount of superoxide dismutase (SOD), an important ROS scavenger. Reactive nitrogen species (RNS), such as nitric oxide (NO) and peroxynitrite (ONOO^−^), are also types of oxygen-related species implicated in neonatal asphyxia. Previous studies reported an association between serum or cerebrospinal fluid (CSF) NO and neonatal asphyxia although the findings are controversial (9–11). NO is generated by activated immune cells, such as neutrophils and macrophages, during an inflammatory reaction (12). ONOO^−^, which is generated by a reaction between NO and •O_2_^−^, leads to protein oxidation, lipid peroxidation, and DNA damage (3). Indeed, plasma nitroalbumin, a potential biological marker of nitrating species generation or nitrative stress, was found to increase significantly in neonates with moderate to severe neonatal encephalopathy a day after birth (13). Thus, nitrative stress is associated with the inflammatory reaction seen in neonatal asphyxia.

Unfortunately, the SOD function in fetuses and neonates is weak. For example, copper and zinc (Cu, Zn)-SOD activity in the cerebral cortex and white matter was remarkably lower in fetuses and neonates than in adults (15% and 50% of the adult value, respectively) because SOD activity increases with increasing gestational age (14–16). On the other hand, birth suddenly imposes considerable oxidative stress on neonates as they transition from a hypoxic uterine environment to normal ambient air. The imbalance between the increased ROS production and weak detoxification ability may cause further oxidative injury to the tissues and organs of neonates at birth. Bilirubin stimulates SOD gene expression but it is insufficient to prevent oxidative stress completely (17). SOD also attenuates cytokine-related reactions in endothelial cells, which play an important role in inflammatory reactions, including neonatal asphyxia. Thus, SOD may be strongly associated with the inhibition of the inflammatory reaction in neonatal asphyxia.

## 3. Inflammation and inflammatory mediators in neonatal asphyxia

Table 1 shows the relationship between neonatal asphyxia and inflammatory mediators. Excessive inflammation exacerbates tissue/organ damage through gene expression and proteins. Elevated cytokines activate various inflammatory immune cells *via* receptors, including toll-like receptors (TLRs), further increasing the production of cytokines and other inflammatory mediators. For instance, the concentration of cytokines, such as interleukin (IL)-1β, IL-6, IL-8, and tumor necrosis factor (TNF)-α, increased to a significantly higher degree in the serum and CSF of asphyxiated neonates than healthy neonates (18–21). Thus, neonatal asphyxia can trigger more potent inflammation as part of a defensive reaction.

**Table 1:** Temporal alteration of inflammatory meiators in asphyxiated neonates.

UB	1–6 h	∼24 h	24 h∼	References
**Increase**				
IL-6	IL-6	IL-6	IL-6	(18–20, 127–130)
	IL-8	IL-8	IL-8	(17, 18, 127)
TNF-α		TNF-α	TNF-α	(19, 128, 131)
		IL-1β	IL-1β	(19)
IL-10	IL-10			(18)
IL-16				(129, 132, 133)
S100B	S100B	S100B	S100B	(128, 135)
GFAP		GFAP	GFAP	(127, 36)
HMGB1	HMGB1			(21, 131)
VEGF			VEGF	(127, 137)
LOX-1				(31)
			GM-CSF	(17)
			NO	(6)
**Decrease**				
VEGF			VEGF	(29)
			IL-6	(20)
			TNF-α	(130)

BDNF, brain-derived neurotrophic factor; GFAP, glial fibrillary acidic protein; GM-CSF, granulocyte-macrophage colony stimulating factor; HMGB1, high- mobility group box 1; IGF-1, insulin-like growth factor 1; IL, interleukin; LOX-1, lectin-like oxidized low-density lipoprotein receptor-1; NO, nitric oxide; TNF-α, tumor necrosis factor-α; UB, umbilical cord blood; VEGF, vascular endothelial growth factor.

### 3.1. IL-6

IL-6 is associated with endothelial cell dysfunction. Increased IL-6 changes endothelial permeability, exacerbating edema of brain or general in asphyxiated neonates (22). Moreover, IL-6 deficiency protects against angiotensin II-induced endothelial dysfunction (23). The IL-6/IL-6 receptor reaction may activate signal transducer and transcription 3 activator (STAT3). In endothelial cells, the IL-6/STAT3 pathway promotes xanthine oxidase, resulting in increased ROS production. In addition, inhibitors of xanthine oxidase or SOD decrease neutrophil recruitment in tissues with hypoxic injury and neutrophil adhesion to endothelial cells (24, 25), with the resulting decrease in IL-6 suppressing the inflammation. IL-6 is also associated with fetal growth retardation (FGR). An *in vitro* study demonstrated that the IL-6/STAT3 signaling pathway induced premature cellular senescence *via* ROS and DNA damage in human fibroblasts (26). IL-6 may cause placental cell senescence, which is associated with detrimental effects, including FGR, preterm spontaneous labor or preterm (27). Chronic stimulation of cells by cytokines causes enough stress to arrest growth or induce senescence. Thus, prolonged hypoxia during the fetal period may contribute to developing FGR *via* IL-6 pathway.

IL-6 has neuroprotective qualities (28) and is associated with manganese (Mn)-SOD, which is encoded by the Mn-SOD gene and localizes in the mitochondrial matrix (29). Mn-SOD removes superoxide anions and ROS (28, 30, 31) while SOD plays a role in controlling the concentration of O2- produced by xanthine oxidase, NADPH oxidase (Nox), and the mitochondria (32). IL-6 binding to IL-6R (forming the IL-6/IL-6R complex) acts as an antioxidant by regulating STAT3-mediated Mn-SOD gene expression although ischemic reperfusion blocks the formation of this compound *via* decreased IL-6R (33). However, a previous study demonstrated that IL-6 therapy restored IL-6R and the IL-6/IL-6R compound, increasing STAT3-mediated Mn-SOD gene expression in a murine model (28). Thus, IL-6 plays a role in protecting neonates from ROS *via* Mn-SOD although a porcine model with FGR had significantly decreased gene expression of Mn-SOD (34). Asphyxiated neonates sometimes show persistent increases in serum CRP within the few first weeks after birth (35–37). IL-6 in these neonates may be produced as a neuroprotective factor, resulting in the persistent increase of IL-6-induced CRP. Thus IL-6 has not only adverse, but also beneficial, effects.

Of all the cytokines, IL-6 has perhaps been the most extensively investigated. Many clinical studies have addressed the relationship between the outcomes of neonatal asphyxia and the concentration of mediators in umbilical cord blood, neonatal blood, and cerebrospinal fluid (CSF) (38–42). A high IL-6 level predicts a poor outcome, such as death or abnormal neurodevelopment. The IL-6 level in neonates with hypoxic-ischemic encephalopathy (HIE) was over 300-fold higher than in non-HIE neonates (38). Serum IL-6 was found to be elevated in neonates with severe asphyxia or meconium aspiration syndrome (20, 43). In asphyxiated neonates, IL-6 production may be triggered by hypoxia, cytokines or ox-LDL (44). ROS also acts as a signaling element to regulate the secretion of proinflammatory cytokines (22),. The IL-6 CSF/plasma ratio in neonates with HIE was higher than in neonates with sepsis or normal neonates (45), suggesting that IL-6 might be produced in the central nervous system, then extravasate into circulating blood. Moreover, the CSF IL-6 level may more accurately predict outcomes than the serum or plasma IL-6 level. However, the biomarkers require patients to be in stable condition and the sampling method to be easy to perform repeatedly. CSF sampling is unsuitable for unstable asphyxiated neonates because it is invasive. On the other hand, the serum or plasma IL-6 level correlates well with outcomes, such as death, HIE severity, neurodevelopment, and the Bayley scale (38–40, 45). The IL-6 concentration in both blood and CSF strongly correlated with outcomes in asphyxiated neonates. Moreover, serum and plasma IL-6 values may reflect the CSF IL-6 value.

### 3.2. Nuclear factor-kappa B (NF-κB)

NF-κB forms a family of transcription factors that play an essential role in the immune inflammatory response. The complex, formed by NF-κB and inhibitor κB (IκB), has an inactive form in cytosol. Oxidative stress activates IκB kinase (IKK) *via* TNF-α, IL-1 or ROS (46). The activated IKK then decomposes IκBα, resulting in decomposition of the complex and translocation of NF-κB into the nucleus where it combines with the DNA to activate the NF-κB pathway. ROS in oxidative stress activate inflammatory cells *via* TLR-4 mediated NF-κB activation (47). Hypoxia-induced-IL-1β also increases the gene expression of NF-κB. Thus, both ROS and hypoxia can activate the NF-κB pathway. The activated NF-κB pathway in turn increases the gene expression of inflammatory mediators and regulates RNS, including iNOS synthesis and NO production, in the neurons of the hippocampus and cortex (48, 49).

One major role of NF-κB is to enhance the inflammatory response by increasing the gene expression of inflammatory mediators, such as TNF-α, IL-1β, and IL-6. ROS-induced oxidative stress activated NF-κB in neurons, astrocytes, microglia, and endothelial cells (50, 51). On the other hand, its activation possibly indicates the mechanism underlying the neuroprotective effect in a murine model of ischemic brain overexpressing Cu,Zn-SOD (52). NF-κB acts not only as a protein transcription factor but also as a regulator of innate immunity *via* toll-like receptors (TLRs) (53, 54). NF-κB may attenuate ROS to promote cellular survival. Thus, increased expression of NF-κB may not always be detrimental to neonates.

Resuscitation with pure oxygen increased NF-κB activation in a murine model (55). In vitro, the NF-κB activation level of peripheral blood mononuclear cells in asphyxiated neonates with neurological sequelae was higher than in non-asphyxiated neonates (56). Indeed, NF-κB orchestrates inflammatory responses. Serum high- mobility group box 1 (HMGB1) also increased in neonatal asphyxia (57). HMGB1, a damage-associated molecular patterns (DAMPs) protein, binds to TLR-4, activating the nuclear factor kappa B (NF-κB) pathway, resulting in an enormous increase in inflammatory mediators (58). However, HMGB1 also play a role in brain development during fetus (59). HMGB1 may act as a mediator during brain development and brain damage. In addition, cell-penetrating, anti-NF-κB peptide treatments administered intranasally attenuated NF-κB signaling, microglia activation, and brain damage in a neonatal, infection-sensitized, HI animal model (60). Anti-NF-κB peptide treatments can regulate robust inflammatory responses.

### 3.3. Hypoxia-inducible factor (HIF) -1

HIF-1, a heterodimer composed of the inducible HIF-1α and constitutive HIF-1β subunits, is tightly regulated by cellular oxygen tension and is essential to surmounting the hypoxic environment in neonates. Hypoxia increases the expression of HIF-1 (61). Increased HIF-1 is associated with ROS production *via* NOS or Nox. HIF-1-induced NOS produces small amounts of ROS in physiological cell signaling. During inflammation, the increase in HIF-1 induces NOS and reduces arginine, leading to increased ONOO^−^ and O2- production (NOS uncoupling). In addition, HIF-1α promotes the production and activation of Nox, an enzymatic factor in ROS production, which includes Nox-1 to 5 and dual oxidase-1 and -2 (62–64). HIF-1-induced Nox promotes ROS production by transferring electrons from NADPH to oxygen. The resulting increase in Nox-derived ROS, then further promotes HIF-1α production (65, 66). Ischemia also activates Nox in immune as well as non-immune cells, such as neurons, vascular endothelial cells, and microglia. In severe hypoxia, accumulated HIF-1α causes necrosis and apoptosis (67) with calcium and calpain and exacerbates cerebral edema by increasing the permeability of the blood-brain barrier (BBB) (67). Movement of the activated immune cells through the BBB promotes an inflammatory immune reaction in the brain involving ROS and cytokines. Thus, HIF-1 may exacerbate neonatal asphyxia by further increasing both ROS and NOS production.

On the other hand, HIF-1 may protect the fetus from hypoxic injury (61). HIF-1α expression is increased in fetuses with physiological hypoxia and is highest in the brain, decreasing slightly with increasing gestational age (68). HIF-1α also increases erythropoietin (EPO) and vascular endothelial growth factor (VEGF). These promote the development of the brain and other organs in the fetus (69). HIF-1 preconditioning before or at birth may help to attenuate hypoxia-related injuries (69, 70). Hypoxia induced mit-ROS inhibits HIF-1α hydroxylation, resulting in HIF-1α accumulation (71). In a postnatal hypoxic state, such as neonatal asphyxia, accumulated HIF-1α binds to HIF-1β subunits, forming a complex that then binds to hypoxia-response elements (HRE) and activates more than 100 genes which help the neonate adapt to the hypoxic environment (61, 72, 73).

Serum HIF-1α was found to increase under conditions of severe asphyxia in accordance with the severity (74). HIF-1α related proteins, such as microRNA (miRNA)-373 (HIF-1α dependent miRNA) and VEGF, also increased in neonatal HIE (74). Downregulation of HIF-1α increased cellular apoptosis in a neonatal HIE rat model (75). Prolonging the HIF-EPO signaling pathway *in vitro* was associated with attenuation of neuronal apoptosis during therapeutic hypothermia (76). Thus, HIF-1 does not always have an adverse effect. Indeed, HIF-1 may increase blood circulation in the brain *via* production of EPO and VEGF, which has an anti-apoptotic and neovascularizing effect, respectively (67). However, serum EPO was higher in HIE neonates than in non-HIE neonates (77) and was associated with poor outcomes, including death and abnormal MRI findings (77). Serum VEGF was lower in neonates with moderate to severe HIE receiving therapeutic hypothermia (77, 78). Whether increased HIF-1 is associated with a good or poor outcome in neonatal asphyxia is still moot although the answer may depend on the HIF-1 concentration involved.

### 3.4. Lectin-like oxidized low-density lipoprotein receptor-1 (LOX-1)

Lectin-like oxidized low-density lipoprotein receptor-1 (LOX-1) is thought to be a biomarker of several diseases, including vascular inflammation and atherosclerosis. LOX-1 is expressed on the surface of the vessel wall cells as a scavenger receptor that mediates the cellular effects of oxidized low-density lipoprotein (OxLDL), resulting in ROS generation (79–81). LOX-1 increases with an increase in OxLDL, free radicals, proinflammatory cytokines, high glucose, and endothelial cell dysfunction and plays a prominent role in neonatal asphyxia (81). Thus, LOX-1 may be a useful biomarker in asphyxiated neonates. Recently, Akamatsu et al. demonstrated that serum LOX-1 was significantly higher in moderately and severely asphyxiated neonates than in normal neonates (82). Ox-LDL/LOX-1 markedly increased VEGF mRNA expression *in vitro* (83). In addition, both ROS and ischemic reperfusion induced LOX-1 in endothelial cells and microglia (79, 84, 85). LOX-1 regulates microglial activation in neonatal HIE (86) and is also associated with ferroptosis, an iron-dependent form of cell death (4, 5), which may be involved in neonatal hypoxic-ischemic brain damage (4, 87). A high LOX-1 level contributes to neuronal apoptosis, breakdown of the BBB, and increased inflammatory mediators and ROS (88–90). Indeed, anti-LOX-1 therapy was found to decrease oxidative stress and inflammation (91, 92); the drug used in the study has the potential to become a novel therapy for neonatal asphyxia.

### 3.5. Toll-like receptors (TLRs)

TLRs, or transmembrane proteins, are a crucial pattern recognition receptor on the surface of both immune cells (e.g., macrophages, neutrophils) and non-immune cells (e.g., cardiac myocytes, hepatocytes, vascular smooth muscle cells) (93). TLR signaling can activate the innate immune system, inducing the initial inflammatory response. Endogenous ligands to TLR include proteins which are actively or passively released by cells. DAMPs, which are potent endogenous ligands and include HMGB1 and heat shock proteins, are an SOS signal transmitted by various cells during hypoxia (94). After tissue injury, they are released extracellularly to activate the immune system and result in a pro-inflammatory cascade. DAMPs activate TLR-2 and TLR-4 signaling after oxidative stress, leading to neuronal apoptosis and ROS production (95).

TLRs are present in both glial cells and neurons and are associated with the pathophysiology of neonatal hypoxia-ischemia. Hypoxia-ischemia increased the expression of TLRs, such as TLR-1, TLR-2, TLR-4, and TLR-7, but decreased TLR-5 and TLR-8 expression in neonatal mouse brains (96). HIF-1α bound to the promoter region of TLR-4 during hypoxia, increasing TLR-4 expression on the surface of macrophages, which peaked at eight hours after hypoxic exposure *in vitro* (97, 98). Furthermore, HIF-1α activated the NF-κB pathway *via* TLR-4 on the surface of microglia during hypoxia, leading to the production of inflammatory mediators, such as TNF-α, IL-1β, and inducible NOS (99). These mediators injure oligodendrocytes and neurons in HIE. In addition, TLR-2 and TLR-4 deficient mice had a smaller infarct size and decreased reperfusion-induced ROS production and leukocyte infiltration (100, 101). Bilirubin scavenged ROS produced by TLR-4-dependent activation of Nox, inhibiting the up-regulation of iNOS (102, 103). In an ovine model of asphyxia, TLR-2 and TLR-4 expression in the cortex and subcortex increased to a significantly higher degree after resuscitation with 100% oxygen than with 21% oxygen (104). ROS then activated inflammatory cells *via* TLR-4-mediated NF-κB activation (47). In asphyxiated neonates, LPS-induced TLR-4 expression in neutrophils increased on day 3 after a hypoxic event (105).

## 4. Immune cells related to inflammation in neonatal asphyxia

### 4.1. Neutrophils

Neutrophils play a prominent role in oxidative tissue/organ injury. Primed neutrophils produce ROS and inflammatory mediators. ROS regulates both apoptotic and necrotic cell death depending on the severity of the oxidative stress (106). First, neutrophils are primed by inflammatory mediators, such as chemokines. The priming times in neutrophils depend on the kind of inflammatory mediator involved. For instance, platelet-activating factor (PAF) primes neutrophils transiently, but granulocyte-macrophage colony-stimulating factor (GM-CSF) prolongs the priming process (107). The PAF level in the CSF of asphyxiated neonates was found to be abnormally high but decreased after head-cooling (107). Serum GM-CSF was significantly higher in neonates with moderate to severe brain injury than in those with mild or no injury 96 h after birth (108). GM-CSF not only extends the preservation of the primed state of neutrophils but also delays neutrophil apoptosis by increasing the stability of Mcl-1 (an anti-apoptosis protein) (109, 110). On consequence, GM-CSF may prolong the production of ROS generated by neutrophils, thereby enhancing tissue injury. Indeed, a high serum GM-CSF level is related to abnormal cranial MRI findings and developmental delay at age 2 years (19). In addition, primed, circulating neutrophils move into sites of hypoxic injury where they accumulate.

Inflammatory mediators, such as IL-6, IL-8, and TNF-α, also have an important role in immune cell extravasation. Neutrophils and other inflammatory cells produce ROS at hypoxic injury sites in addition to secreting inflammatory mediators (111, 112). Moreover, inflammatory mediators may promote transmigration of neutrophils over endothelial cells and the BBB, prolong cell survival, and increase degranulation (113, 114). Interestingly, the accumulated time course of inflammatory cells was consistent with increased ROS generation and tissue/organ injury (115). In particular, IL-8 is a potent chemokine which enhances neutrophil functions in inflammatory reactions, including their migration from circulating blood, chemotaxis to inflammation sites, activation in areas of inflammation, and priming of immune cells (116). Increased IL-8 in the serum and cerebrospinal fluid of asphyxiated neonates was associated with poor outcomes (19, 20). IL-8 does not directly activate Nox but rather phosphorylates Nox components and moves to the lipid rafts of neutrophils, leading to extracellular ROS generation (109, 117).

### 4.2. Endothelial cells

The collapse of endothelial cell function directly induces an accumulation of inflammatory cells in extravascular, hypoxic injury sites. Immediately after ischemia, small amounts of ROS, such as xanthine oxidase-derived superoxide anion, cause endothelial cell barrier dysfunction (increased permeability) and increase adhesion molecule expression on the surface of endothelial cells and neutrophils. This promotes the extravasation of circulating immune cells. ROS further promotes the gene expression of proinflammatory proteins (e.g., cytokines and adhesion molecules) in endothelial cells and the BBB (118) and triggers an increase in matrix metalloproteinases downstream, causing a breakdown in the BBB (119). SOD mimetics reduced production of pro-inflammatory cytokines (e.g., TNF-α, IL-1β, IL-6) as well as of cell adhesion molecule expression on endothelial cells, decreasing neutrophil extravasation (120–122). In endothelial cells, SOD is associated with decreased ROS production and cell adhesion molecule expression *via* cytokine-related processes.

Adhesion molecules on the endothelial cell surface are important for enabling the migration of inflammatory cells to affected areas. IL-6 induces the expression of adhesion molecules, including intercellular adhesion molecule (ICAM)-1, vascular cell adhesion molecule (VCAM)-1, and e-selectin. Further, oxidative stress and RAS-related C3 botulinus toxin substrate-1 (Rac1) activation increased ICAM-1 expression *via* the IL-6/STAT3 pathway in endothelial cells, increasing inflammatory cell infiltration (123). On the other hand, IL-6 can directly reduce eNOS expression (124). IL-6 dose-dependently reduced eNOS expression in cultured endothelial cells *in vitro* (125) and suppressed the IL-6/STAT3 pathway, resulting in decreased ICAM-1 expression (126). In addition, ROS promotes coagulation in endothelial cells although NO inhibits platelet aggregation (127). VCAM-1 is expressed on the endothelial cell surface and promotes neutrophil transmigration; this priming produces ROS. On the other hand, cross-linking of VCAM-1 activates calcium flux and Rac-1 in endothelial cells (128), which in turn activates Nox and results in ROS generation. The VCAM-1 signaling *via* ROS mediates changes in actin in endothelial cells and leads to the creation of open intercellular passageways *via* changes in endothelial cell structure (129), enabling many neutrophils promptly to transmigrate to sites of injury. Antioxidants, such as bilirubin and vitamin E, were found to inhibit VCAM-1-dependent neutrophil recruitment both *in vivo* and *in vitro* (130). VCAM-1 blockade inhibited leukocyte recruitment and IR injuries in the liver (129, 131).

## 5. Treatment

### 5.1. Therapeutic hypothermia

Therapeutic hypothermia, including cooling of the body or head, influences inflammatory mediator levels in neonatal asphyxia (Table 2). Asphyxiated neonates receiving therapeutic hypothermia demonstrated a temporary increase in serum cytokines [e.g., IL-6, IL-8, IL-10, granulocyte-colony stimulating factor, monocyte chemoattractant protein (MCP)-1] and a temporary decrease in serum cytokines [e.g., VEGF, macrophage inflammatory proteins (MIP-1α)] (78, 132). Thus, hypothermia can mitigate the inflammatory response in neonatal asphyxia.

**Table 2:** Temporary changes in inflammatory mediators in asphyxiated neonates treated with therapeutic hypothermia.

1–6 h	∼24 h	24 h∼	References
**Increase**			
VEGF	VEGF	VEGF	(138, 139)
	GFAP	GFAP	(117, 138)
	G-CSF	GM-CSF	(30, 76)
		RANTES	(138)
**Decrease**			
IL-6		IL-6	(139)
	VEGF		(30, 117)
		IL-8	(140)
		IL-1RA, IL-10	(141)
		S100B	(134)
		RANTES	(76)

G-CSF, granulocyte colony stimulating factor; GFAP, glial fibrillary acidic protein; GM-CSF, granulocyte-macrophage colony stimulating factor; IL, interleukin; IL-1RA, Interleukin-1 receptor antagonist; RANTES, regulated upon activation, normal T cell expressed and secreted; VEGF, vascular endothelial growth factor.

Serum granulocyte colony stimulating factor (G-CSF) markedly increased and remained high in severe neonatal asphyxia treated with head cooling (78). G-CSF may mediate anti-apoptosis in neurons (133, 134), and high enough quantities may prevent excessive neuronal death due to hypoxia in severe neonatal asphyxia (135). Brain edema, inflammation, and disruption of the BBB were also mitigated by G-CSF administration (133). On the other hand, VEGF, an HIF-1 related mediator, decreased and remained low (78, 136). VEGF acts as an enhancer of BBB permeability and may cause cerebral edema (137); a high VEGF level may thus exacerbate pre-existing edema (137). Lowering VEGF in severely asphyxiated neonates using head cooling might prevent cerebral edema even in the presence of severe hypoxia. However, decreased VEGF is reportedly associated with poor outcomes, including death and abnormal MRI findings (77). VEGF may be a key mediator, and further research is needed to clarify its association with neonatal asphyxia.

### 5.2. Erythropoietin

EPO, an HIF-1 related mediator, has anti-apoptotic, anti-inflammatory, anti-oxidative, and anti-excitotoxic effects. Its therapeutic efficacy is currently being debated; previous studies have investigated the anti-apoptotic effect of EPO during hypothermia, and other, similar studies are currently being conducted (11, 138–141). However, a recent, multicentric, double-blind, randomized, placebo-controlled trial found that EPO had no significant effect on mortality or neurodevelopmental impairment in a large cohort of asphyxiated neonates and was in fact associated with a high rate of serious adverse events (142). Further studies on the safety, effects, and viability of EPO therapy are clearly needed. At present, many studies aiming to develop medications for use with hypothermia to treat neonatal asphyxia are being conducted (Table 3).

**Table 3:** Unreported or ongoing clinical trial of pharmacotherapy for neonatal asphyxia.

Drugs	Title	Interventions	Subjects	Phase	Country	Status	Number
Erythropoietin	Erythropoietin for Neonatal Encephalopathy in LMIC (EMBRACE Trial)	Erythropoietin monotherapy	Neonatal encephalopathy	Phase III	Bangladesh, India, Sri Lanka	Not yet recruiting	NCT05395195
	Neonatal Erythropoietin And Therapeutic Hypothermia Outcomes in Newborn Brain Injury (NEATO)	1,000/kg/dose × 5 doses	Cooled infants with HIE	Phase I/II	USA	Completed	NCT01913340
	Neurological Outcome After Erythropoietin Treatment for Neonatal Encephalopathy	Either 300 U/kg or 500 U/kg, subcutaneously the first time and then intravenously every other day for 2 weeks.	HIE	Phase I/II	China	Completed	NCT00808704
	Neonatal Erythropoietin in Asphyxiated Term Newborns (NEAT)	250, 500, 1,000, or 2,500 U/kg/dose × 6 doses	Cooled infants with HIE	Phase I	USA	Completed	NCT00719407
	Efficacy of Erythropoietin to Improve Survival and Neurological Outcome in Hypoxic Ischemic Encephalopathy (Neurepo)	1,000 to 1,500 U/kg/dose, 3 dose every 24 h	Cooled infants with HIE	Phase III	France	Completed	NCT01732146
	Neuroprotective Role of Erythropoietin in Perinatal Asphyxia	500 units/kg/day every other day for 5 doses	Perinatal Asphyxia	Phase II/III	India	Completed	NCT02002039
Darbepoetin alfa	Darbepoetin in Neonatal Encephalopathy Trial (EDEN)	10 μg/kg IV, 2 doses following cooling therapy.	Cooled infants with HIE	Phase II	UK	Recruiting	NCT04432662
	Mild Encephalopathy in the Newborn Treated With Darbepoetin (MEND)	10 μg/kg/dose IV, one dose at <24 h of age	Mild HIE	Phase II	USA	Completed	NCT03071861
	Darbe Administration in Newborns Undergoing Cooling for Encephalopathy (DANCE)	2 or 10 μg/kg/dose IV, 2 dose, within 12 h and at 7 days old.	Cooled infants with HIE	Phase I/II	USA	Completed	NCT01471015
Epoetin alfa	Erythropoietin for Hypoxic Ischaemic Encephalopathy in Newborns (PAEAN)	1,000 IU/kg, IV, on Days 1, 2, 3, 5 and 7 of age	Cooled infants with HIE	Phase III	Australia	Active, not recruiting	NCT03079167
Melatonin	Melatonin as a Neuroprotective Therapy in Neonates With HIE Undergoing Hypothermia	0.5, 3, or 5 mg/kg enteral dose	Cooled infants with HIE	Early Phase 1	USA	Recruiting	NCT02621944
	Use of Melatonin for Neuroprotection in Asphyxiated Newborns (MELPRO)	10 mg/kg, 5 daily enteral doses	Cooled infants with HIE	Not Applicable	Italy	Recruiting	NCT03806816
	Melatonin for Neuroprotection Following Perinatal Asphyxia	10 mg/kg daily, 5 doses in total	Cooled infants with HIE	Phase I/II	Egypt	Completed	NCT02071160
Magnesium sulfate	Hypothermia Enhanced by Magnesium Sulphate (Hemen)	250 mg/kg doses, 3 doses in total	Cooled infants with HIE	Phase II/III	Poland	Completed	NCT02499393
	Beneficial Effect of Intravenous Magnesium Sulphate in Term Neonates With Hypoxic Ischemic Encephalopathy (HIE)	250 mg/kg/dose, 3 doses within postnatal 6 h, 24 h, and 48 h	HIE	Phase II	Pakistan	Completed	NCT04705142
	Magnesium Sulphate in Perinatal Asphyxia (Magsulf)	250 mg/kg, every 24 h starting at postnatal 6 h	Moderate to severe HIE	Phase III	India	Completed	NCT00553072
Xenon	Xenon and Cooling Therapy in Babies at High Risk of Brain Injury Following Poor Condition at Birth (CoolXenon2)	Xenon gas 50% for 18 h	Cooled infants with HIE	Phase I/II	UK	Completed	NCT01545271
	Xenon and Cooling Therapy in Babies at High Risk of Brain Injury Following Poor Condition at Birth (CoolXenon3)	Xenon gasv50% for 18 h	Cooled infants with HIE	Phase II	UK	Completed	NCT02071394
Stem cells	Neonatal Hypoxic Ischemic Encephalopathy: Safety and Feasibility Study of a Curative Treatment With Autologous Cord Blood Stem Cells	5.107/kg injection of autologous mononuclear cells from umbilical cord blood	Cooled infants with HIE	Phase I/II	France	Recruiting	NCT02881970
	A Clinical Trial to Determine the Safety and Efficacy of Hope Biosciences Autologous Mesenchymal Stem Cell Therapy for the Treatment of Traumatic Brain Injury and Hypoxic-Ischemic Encephalopathy	3 infusions over 6 weeks at 14-day intervals	Traumatic brain injury	Phase I/II	USA	Active, not recruiting	NCT04063215
	Study of hCT-MSC in Newborn Infants With Moderate or Severe HIE	Single dose within postnatal 48 h	Cooled infants with HIE	Phase I	USA	Completed	NCT03635450
Cord blood cells	Autologous Cord Blood Cells for Brain Injury in Term Newborns	Infant's own umbilical cord blood	HIE	Phase I	Singapore	Completed	NCT01649648
	Neuroprotective Effect of Autologous Cord Blood Combined With Therapeutic Hypothermia Following Neonatal Encephalopathy	Aliquots during postnatal 3 days	Cooled infants with HIE	Phase I/II	China	Recruiting	NCT02551003

HIE, hypoxic-ischemic encephalopathy; IV, intravenous; hCT-MSC, human umbilical cord tissue-derived mesenchymal stromal cells.

### 5.3. Melatonin

Melatonin has potential as a treatment for NA in combination with therapeutic hypothermia. Melatonin acts as an antioxidant, anti-inflammatory mediator, and antiapoptotic agent (143); it not only directly neutralizes ROS and RNS, but also reduces NF-κB translocation and proinflammatory cytokine production (144). Melatonin can also freely cross the placenta and BBB because of its low molecular weight (232.28 g/mol). A pharmacokinetic study of enterally administered melatonin in neonates treated with hypothermia (33.5°C) showed that its half-life (mean 50.9 h) and clearance (mean 0.046 L/h/kg) were greater while its distribution volume (mean 1.80 L/kg) was smaller than in adults (145). Moreover, melatonin is safe. A randomized trial of a combination of melatonin and therapeutic hypothermia reduced oxidative stress and improved neurodevelopmental outcomes in neonates with moderate to severe HIE at age 6 months (145). Thus, melatonin has the potential to be a viable treatment for NA.

### 5.4. Magnesium sulfate

Magnesium has immunomodulatory activity (146). Therefore, magnesium sulfate was examined for its potential to improve outcomes in neonatal asphyxia by inhibiting cellular calcium influx and excitatory amino acid release in neurons *via* blockade of the NMDA-receptor channel, which has a magnesium-dependent calcium gate (147, 148). Magnesium deficiency opens the NMDA-receptor channel and leads to an influx of calcium. The increased, intracellular calcium ions stimulate production of substance *P*, which promotes pro-inflammatory cytokines (149), which prime phagocytes, such as macrophages, and increase ROS production (150). Thus, magnesium deficiency is linked to both increased inflammation and oxidative stress (150). On the other hand, magnesium administration rapidly increased the intracellular magnesium level (146), leading to an increased intracellular IκBα concentration, which inhibited NF-κB activation (146). Consequently, magnesium may decrease inflammation and oxidative stress *via* reduced NF-κB. Interestingly, it was reported that suppressing the NMDA-receptor *via* magnesium blockade was essential to forming long-term memory in Drosophila (151). Magnesium therapy may inhibit or delay ischemic cell death and decrease neurodevelopmental deficits. A previous RCT showed that magnesium improved short-term outcomes, such as cranial imaging findings, oral feeding establishment, and neurological outcomes at discharge (152, 153). A few RCT also reported that postnatal magnesium sulfate improved developmental outcomes, but the studies did not enroll a large cohort (154). A recent review and meta-analysis failed to find any long-term benefits but found instead an increased trend in mortality in patients receiving magnesium (155, 156). Research into the effect of the combination therapy consisting of hypothermia and magnesium sulfate in neonates with HIE is still ongoing, but results indicating favorable, long-term outcomes are expected like other treatments (Table 3).

### 5.5. Xenon

Xenon, a noble gas, was first used as a general anesthetic with few adverse effects (157). Xenon's neuro-protective qualities attracted a great deal of attention (158). The molecular mechanism of Xenon's activity involves inhibiting the NMDA receptor, stabilizing the membrane potential, inhibiting Bax-induced apoptosis, increasing the anti-apoptosis genes, Bcl-x-L and Bcl 2, and inhibiting activated inflammatory cells (e.g., macrophage) (159). Many preclinical studies using animal models reported that a combination of hypothermia and inhaled xenon had neuroprotective effects in neonatal HIE (160–162). However, in the sole human study to date, inhaled Xenon failed to improve clinical signs and MRI findings (TOBY-Xe) (163). One problem with this study was that xenon inhalation was begun after postnatal hour 6, which is outside the therapeutic window. This delay may have affected the results. In the future, more human studies are needed to assess the effects of inhaled xenon within the first six hours of life.

### 5.6. Hydrogen (H_2_)

Hydrogen gas (H_2_), an expected novel therapeutic agent, selectively reduces •OH as a radical scavenger (164). In addition, H_2_ decreases inflammatory cytokines, including TNF-α and NF-κB, as an anti-inflammatory agent (165, 166) and induces anti-apoptotic molecules as an anti-apoptotic agent (167). Thus, H_2_ may be associated with preventing lung or brain from ROS or inflammations. Indeed, H_2_ prevented brain injuries in an adult rat model of ischemic reperfusion (168). The combination between H_2_ inhalation and hypothermia (33.5 ± 0.5 °C) in a neonatal pig model of hypoxia-ischemia improved short-term neurological outcomes, such as walking (169). Moreover, it is suitable that the cost of H_2_ is lower though that of Xenon is higher for using in clinical practices. Thus, the clinical use of H_2_ is expected as a novel therapy for neonatal HIE. However, there is still no human studies in neonates though some human studies are conducted in adults, such as coronavirus disease 2019 (COVID-19) (NCT04378712).

### 5.7. Stem cell therapy

Stem cells for neonatal therapy can be derived from autologous umbilical cord blood (umbilical cord blood and Wharton's jelly) or amniotic fluid. There are many kinds of stem cells, such as mesenchymal stem cells (MSCs) and hematopoietic stem cells. These stem cells are capable not only of multipotent differentiation, but also of inhibiting inflammation and oxidative stress, reducing apoptosis, and decreasing mitochondrial dysfunction (170). MSCs, which are commonly used in neonatal animal HIE studies, are characterized by stronger resistance to oxidative stress-induced death and higher activity of both SOD and catalase, which result in reducing oxidative stress (171). MSCs also decrease proinflammatory factors, secrete anti-inflammatory factors, such as IL-10, and inhibit the proliferation and activation of inflammatory cells in the injured cortex (172, 173). Interestingly, MSCs rescued neurons and endothelial cells from dysfunction and apoptosis through tunneling nanotube-mediated mitochondrial transfer (174, 175). In addition, MSCs increased the number of neurons in the hippocampus and improved neurogenic and developmental outcomes, such as cognitive, behavioral, and sensorimotor functions (176–178). In neonatal animal models, other stem cells also demonstrated benefits. For instance, hematopoietic stem cells increased brain blood flow and the vessel diameter, and endothelial progenitor cells decreased neuronal inflammation and cell apoptosis (179, 180).

Indeed, many preclinical studies have demonstrated the effects of stem cell therapy. Phase I studies proved the feasibility and safety of stem cell therapy (181–183). Stem cell therapy for neonatal HIE is expected to confer antioxidant, anti-inflammatory, and anti-apoptosis effects. However, at present, no human study has demonstrated the efficacy of stem cell therapy for neonatal HIE (184). Engraftment of stem cells to sites of injury can induce multipotent differentiation and paracrine growth or anti-inflammatory factors. However, MSCs engraftment has not yet been successful (185). The results of many, currently ongoing clinical trials using MSCs, muse cells, and other cell types await publication (184, 186).

## 6. Summary

**Figure 1:**
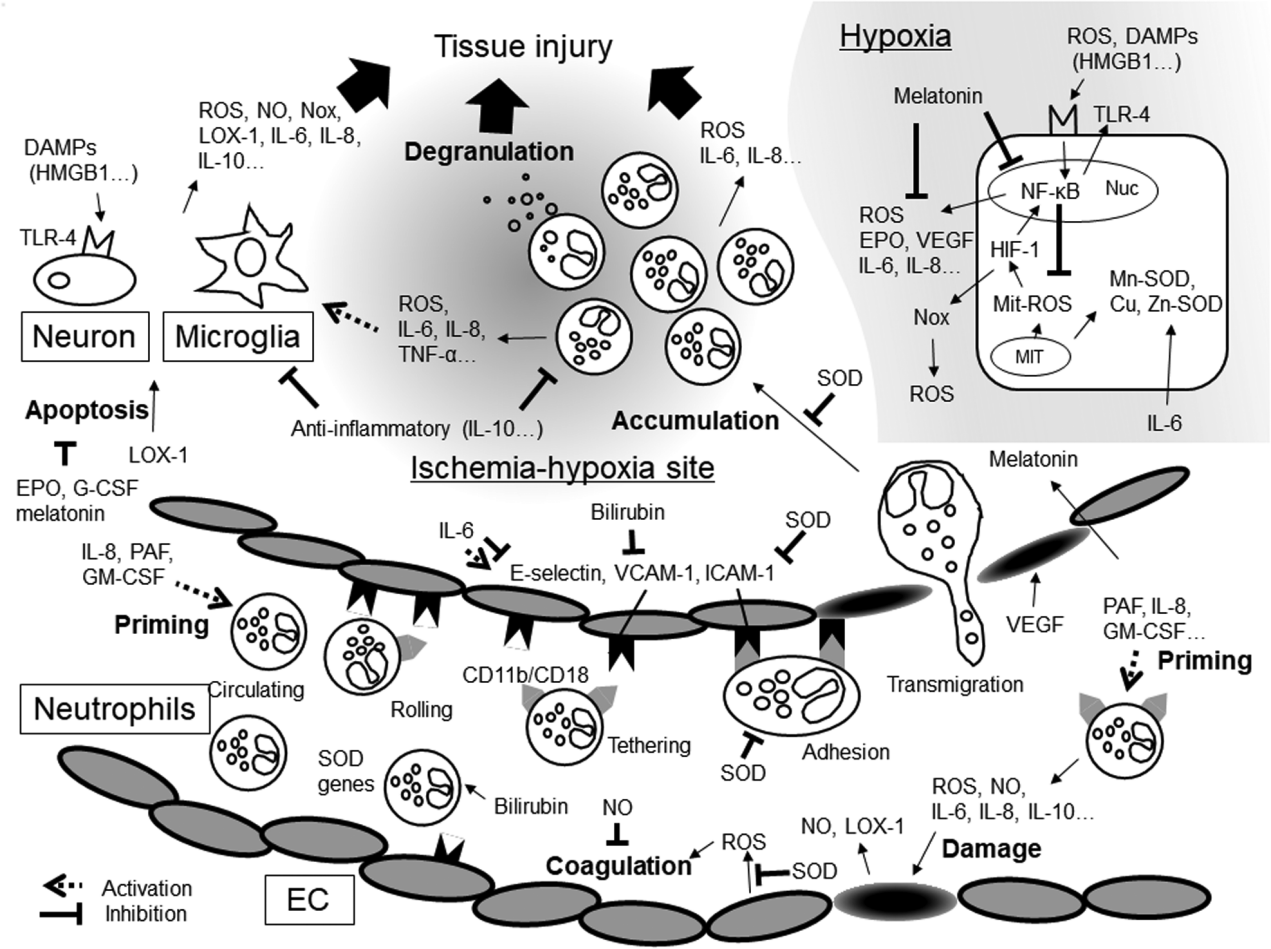
Inflammatory mediators and oxidative stresses in hypoxic-ischemia. DAMPs, damage-associated molecular patterns; EC, endothelial cells; EPO, erythropoietin; G-CSF, granulocyte-macrophage colony stimulating factor; GM-CSF, granulocyte-macrophage colony stimulating factor; HIF-1, hypoxia-inducible factors-1; HMGB1, high- mobility group box 1; ICAM-1, intercellular adhesion molecule; IL, interleukin; LOX-1, lectin-like oxidized low-density lipoprotein receptor-1; MIT, mitochondria; NF-κB, Nuclear factor-kappa B; NO, nitric oxide; Nox, NADPH oxidase; Nuc, nuclear cell; PAF, platelet-activating factor; ROS, reactive oxygen species; SOD, superoxide dismutase; TLR-4, Toll-like receptors-4; TNF-α, tumor necrosis factor-α; VCAM-1, vascular cell adhesion molecule; VEGF, vascular endothelial growth factor.

In NA, inflammatory mediators may promote ROS production and enhance ROS function (Figure 1). ROS can also promote inflammatory mediator production and activate inflammatory cells. Therefore, a novel therapy capable of regulating ROS production and controlling the inflammatory response is much to be desired. However, measuring temporary changes in inflammatory mediator levels soon after birth is challenging. First, the precise timing of NA onset *in utero* cannot be determined although inflammatory mediators may demonstrate drastic, albeit temporary, changes in quantity depending on the time from NA onset. Second, cytokines have pleiotropy and redundancy. However, we can know the timing of hypothermia therapy start. In the future, investigating temporary fluctuations in the level of inflammatory mediators from the start time may lead to the development of a novel, effective therapy.
